# Sertraline-induced 5-HT dysregulation in mouse cardiomyocytes and the impact on calcium handling

**DOI:** 10.1152/ajpheart.00692.2023

**Published:** 2024-10-18

**Authors:** Yongjun Lu, Elizabeth Kenkel, Kathy Zimmerman, Robert M. Weiss, Robert D. Roghair, Sarah E. Haskell

**Affiliations:** ^1^Division of Pediatric Critical Care, Department of Pediatrics, University of Iowa Carver College of Medicine, Iowa City, Iowa, United States; ^2^Division of Neonatology, Department of Pediatrics, University of Iowa Carver College of Medicine, Iowa City, Iowa, United States; ^3^Bristol-Myers Squibb, Seattle, Washington, United States; ^4^Division of Cardiology, Department of Internal Medicine, University of Iowa Carver College of Medicine, Iowa City, Iowa, United States; ^5^Cardiology Section, Department of Veterans Affairs Medical Center, Iowa City, Iowa, United States

**Keywords:** cardiovascular development, cardiovascular disease, depression during pregnancy, selective serotonin reuptake inhibitors

## Abstract

Selective serotonin reuptake inhibitors (SSRIs) are prescribed in 15% of pregnancies in the United States for depression. Maternal use of SSRIs has been linked to an increased risk of congenital heart defects, but the exact mechanism of pathogenesis is unknown. SSRIs, including sertraline, are permeable to the placenta and can produce direct fetal exposure. Previously, we have shown decreased cardiomyocyte proliferation, left ventricle size, and cardiac expression of the serotonin receptor 5-HT_2B_ in the offspring of mice exposed to the SSRI sertraline relative to the offspring of saline-exposed mice. Using a mouse model of in utero plus neonatal sertraline exposure, we observed lengthened peak-to-peak time of calcium oscillation (saline 784 ± 76 ms; sertraline 1,121 ± 130 ms, *P* < 0.001) and decreased expression of critical genes in calcium regulation. We also observed significant upregulation of specific microRNAs (miRNAs) that modulate serotonin signaling in neonatal cardiac tissues (*Slc6a4: miR-223-5p, miR-92a-2-5p, miR-182-5p; Htr2a: miR-34b-5p, miR-182-5p; Htr2b: miR-223-5p, miR-92a-2-5p, miR-337-5p*) (*P* < 0.05) with corresponding levels of the target mRNAs downregulated (*Slc6a4* 0.73 ± 0.05; *Htr2a* 0.67 ± 0.04; *Htr2b* 0.72 ± 0.03; all *P* < 0.01), resulting in decreased production of the cognate proteins. Adult mice at 10 wk showed altered cardiac parameters including decreased heart rates in males (saline 683 ± 8 vs. sertraline 666 ± 6 beats/min, *P* < 0.05) and ejection fraction in females (saline 83.9 ± 0.6% vs. sertraline 80.6 ± 1.1%, *P* < 0.05). These findings raise the question of whether sertraline exposure during development may increase the potential risk for cardiac disease when subjected to stress.

**NEW & NOTEWORTHY** Sertraline exposure during development decreased the expression of critical genes in calcium regulation and lengthened periods in calcium oscillation in neonatal cardiomyocytes. Sertraline upregulated specific microRNAs that may modulate serotonin signaling in neonatal cardiac tissues, which corresponded with a decrease in the levels of the corresponding target mRNAs. Although the echocardiograms in our adult mice suggest a mild phenotype associated with sertraline exposure, these upregulated microRNAs (miRNAs) have been linked to adult cardiovascular disease and heart failure.

## INTRODUCTION

The prescription of antidepressants to treat clinical depression in pregnant women has dramatically increased over the past decade. According to a report from the Centers for Disease Control and Prevention, it is now estimated that ∼15% of pregnant women (one million) in the United States take a selective serotonin reuptake inhibitor (SSRI) during pregnancy (1, 2). SSRIs, including sertraline, are the most commonly prescribed antidepressants. In the fall of 2005, GlaxoSmithKline called attention to safety data indicating an increased risk of congenital malformations among infants exposed during organogenesis to the SSRI, paroxetine (3). Shortly thereafter, the US Food and Drug Administration (FDA) posted a similar warning on its website. Congenital heart disease occurs in ∼1% of live births and several investigations have linked the use of SSRIs to an elevated rate of congenital heart disease, but considerable controversy remains as it is difficult to separate the drug effects from that of the underlying maternal depression (4–8). In a recent systematic review of meta-analyses, analyses of individual SSRIs and congenital heart disease demonstrated increased risks with odds ratios of 1.57 [95% confidence interval (CI) 1.25–1.97], 1.36 (95% CI 1.08–1.72), and 1.29 (95% CI 1.14–1.45) for paroxetine, fluoxetine, and sertraline, respectively (8).

Serotonin (5-hydroxytryptamine, or 5-HT) is a signaling molecule regulating a variety of cellular processes (cell migration, differentiation, and proliferation) that are involved in the development of the mammalian heart. In addition, circulating 5-HT can impact heart rate, contraction, fibrosis, coronary constriction, cardiac conduction, and thrombosis. 5-HT is produced in the nervous system and gut and then transported into the heart via platelets but is generated directly in cardiac cells as well (9, 10). Sertraline inhibits the serotonin transporter (SERT, or SLC6A4) preventing 5-HT reuptake. As a 5-HT uptake blocker, sertraline acutely increases extracellular 5-HT; however, concentrations of 5-HT become reduced with long-term sertraline exposure because of autoinhibitory feedback (11). SERT is a member of the neurotransmitter-sodium symporter family, a member of the monoamine subfamily belonging to the SLC6 transporter family (12). Sertraline has a selective affinity to SERT at 0.29 nM (13). SSRIs cross the placenta (14, 15), so it is plausible that maternal SSRI exposure could affect fetal heart development by altering 5-HT signaling in fetal cardiac cells.

Animal models are essential to examine the effects of SSRI exposure on cardiovascular outcomes, both in adult mice and during pregnancy and development. Lassen et al. (16) observed amelioration of pathologic left ventricular enlargement after ischemia-reperfusion in adult rats treated with paroxetine. Hooper et al. (17) reported that sertraline exposure constricts the mouse ductus arteriosus in utero. Our previous animal studies showed that perinatal exposure to sertraline caused smaller left ventricular dimensions and decreased stroke volumes in adult mice (18, 19), and reduced cardiomyocyte number and lower cardiac expression of *Htr2b,* the gene encoding the 5-HT_2B_ receptor, in neonatal mice and zebrafish embryos (19, 20). Mice lacking peripheral 5-HT or carrying loss-of-function mutations in the 5-HT receptor gene (*Htr2b*) display cardiac insufficiency and dilated cardiomyopathy resulting in embryonic and neonatal death (9, 21, 22), as do mice with mutations in the serotonin transporter (23–25). In surviving adult *Htr2b* null mice, left ventricular dilation and decreased systolic function were present (22). Although we have not observed congenital heart defects in our mouse model of sertraline exposure thus far, we have seen important changes in cardiomyocyte development that impact the cardiovascular performance of adult mice that often appear asymptomatic at rest (18, 19).

Outside of cardiac defects, SSRIs have also been shown to affect calcium signaling (26–30). Calcium is essential for normal heart function, and calcium dysregulation is one of the hallmarks of a failing heart (31). Calcium cycling in cardiomyocytes is an integral process involving the dynamic interplay of various proteins, ultimately controlling muscle contraction and relaxation. In brief, muscle thin filament consists of actin, troponins (troponins C, I, and T), and tropomyosin. Calcium binding to troponin C will trigger significant conformational changes in troponin I and troponin T, and in tropomyosin to allow actin-myosin interactions. Specifically, calcium release from the sarcoplasmic reticulum initiates contraction, whereas its removal from the cytoplasm allows for muscle relaxation. SSRIs have been linked to the inhibition of ion channels, inhibition of the L-type calcium current in cardiomyocytes, and depressant effects on cardiac cells (32–34). Importantly, alterations in serotonin signaling have been associated with changes in contractile function and excitation-contraction coupling, which can lead to cardiomyopathy and arrhythmias (9, 21, 22, 35, 36).

Because of the frequent and increasing use of SSRIs during pregnancy, it is critical to better understand how SSRIs impact development and beyond birth. Recent reviews highlight the impact of perinatal SSRI exposure on body weight, neurodevelopment, and behavioral abnormalities using animal models of SSRI exposure (37, 38). Importantly, these studies are in the absence of depression as there are unique challenges to modeling depression in animals (39). We and others have previously demonstrated the importance of the serotonin signaling pathway in heart development (9, 16–25). Using a perinatal SSRI animal exposure model, we seek a deeper mechanistic understanding of the long-term effects of disrupted 5-HT signaling by sertraline on the heart. In this study, we hypothesized that sertraline exposure would, via SLC6A4, cause microRNAs (miRNAs) to epigenetically regulate target genes in the serotonin signaling pathway, resulting in abnormal cardiac development and function.

## MATERIALS AND METHODS

### Animals and Sertraline Exposure Model

All experimental protocols and procedures fully met the standards set forth within the regulations of the Animal Welfare Act and the National Institutes of Health Guide for the Care and Use of Laboratory Animals and were approved by the Institutional Animal Care and Use Committee of the University of Iowa.

**Figure 1.**
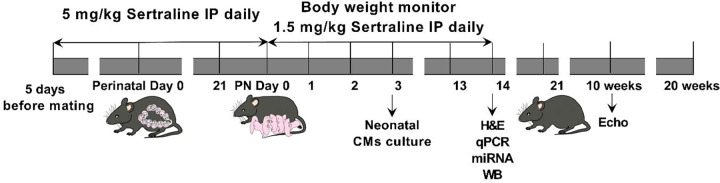
Sertraline exposure model. C57BL/6J mice at the age of 10-wk old were used. The female mice either received saline or sertraline (intraperitoneal) each day for the 5 days before mating with males, and daily throughout pregnancy to the time of delivery. Pups were weighed immediately prior to daily administration of saline or sertraline from postnatal (PN) age 0 to 14 days. Pups of *PN day 3* were euthanized for cardiomyocyte (CM) dissociation and culture. Pups of *PN day 14* were euthanized for tissue analysis. Pups raised to adults were subjected to echocardiography at 10 wk of age. H&E, hematoxyline-eosin; miRNA, microRNA analysis; WB, Western blot.

The sertraline exposure model was described in our previous paper and is summarized schematically in Fig. 1 (19). Briefly, groups of C57BL/6J female mice, numbering up to five individuals each, were accommodated together, whereas stud males were housed individually in cages that were both individually ventilated and specified pathogen-free. The cages were equipped with absorbent bedding and a cardboard tube. The females received either intraperitoneal saline (10 mL/kg/day) or sertraline (5 mg/kg/day) for each of the 5 days prior to mating. After a period of 5 days, the female mouse underwent mating in the afternoon with a male mouse. The following morning, successful mating was identified by the presence of a whitish copulation plug in the female’s vaginal opening, denoted as the first day for gestation day calculations. Daily intraperitoneal injections of sertraline solution or saline were administered throughout pregnancy to the time of delivery. To maintain a similar exposure throughout the period of cardiac proliferation in mice (40, 41), pups received sertraline 1.5 mg/kg/day or saline 3 mL/kg/day, approximately one-third of the maternal dose to reflect placental transfer during pregnancy (14), on *postnatal* (*PN*) *days 0*–*14*. The neonatal exposure matched the prenatal exposure. The sertraline dose used was determined previously from murine body surface area, average body weight and height of women in the third trimester, oral bioavailability, and placental transfer (42). Based on these parameters, a typical human clinical low-dose therapy of 100 mg/day translates to ∼5 mg/kg/day for mice. At this dosing, the sertraline-exposed mouse pups on *PN day 14* had plasma levels of less than 10 ng/mL (43), which is equivalent to umbilical levels of sertraline in humans (14).

The mice were observed every 12 h, and the weight of the newborn pups was measured just before daily administration until they were euthanized for subsequent experiments. Weights were recorded at the time of weaning (*postnatal day 21*) and at 10 wk of age. The lactating dams were kept with their respective litters until the offspring were weaned on the 21st day after birth. For sampling, the mice were euthanized using CO_2_ inhalation followed by cervical dislocation, conducted in accordance with institutional guidelines for humane animal treatment, and the hearts were rapidly excised and frozen in liquid nitrogen and stored at −80°C or placed in 1% formalin, or in 200 µL of TRIzol regent (Invitrogen).

### Cardiomyocytes Dissociation and Culture

Cardiomyocytes (CMs) were isolated from male and female mice and pooled. The mouse CMs were prepared by using kits according to the protocol of the manufacturer (Miltenyi Biotec) and as previously described (19). In brief, the heart of each neonatal mouse was harvested at *postnatal day 3* and transferred into a 10-cm dish containing phosphate-buffered saline (PBS). The remaining blood was released out of the heart carefully and the vessels and remaining connective tissue were cut away from the ventricles as cleanly as possible. The hearts were dissociated into single-cell suspensions by using a neonatal mouse heart dissociation kit according to the protocol of the manufacturer (Miltenyi Biotec). First, the neonatal hearts were treated enzymatically to degrade the cardiac extracellular matrix; this treatment maintains the structural integrity of tissues. The treated hearts were then dissociated mechanically using the gentleMACS Dissociator (Miltenyi Biotech). A 70-mm strainer was used to remove larger particles from the suspension. Erythrocytes in the cell suspension were removed by lysis, using a commercial Red Blood Cell Lysis Solution (Miltenyi Biotech). Subsequently, using the Neonatal Cardiomyocyte Isolation Kit (Miltenyi Biotech), mouse CMs were isolated by binding to antibody-conjugated paramagnetic beads (recognizing CM epitopes) in MS Columns exposed to a magnetic field (Miltenyi Biotech). Following elution, the purified CMs were plated at a density of 2.5 × 10^6^ per well in 12-well dishes pre-coated with Collagen Type I Rat Tail (Corning). A revised culture medium recipe was used for the culture of the CMs: 65% Dulbecco’s modified Eagle’s medium (DMEM) + 19% medium 199 (M-199) + 10% horse serum + 5% fetal bovine serum (FBS), 1% penicillin-streptomycin, all purchased from Thermo Fisher Scientific. The calcium ion concentration, ∼1.2 mM in the physiological range, was achieved using DMEM. Within 24-h postseeding, the cultured cells displayed discernible beating.

### Calcium Imaging and Spontaneous Calcium Transients

We assessed the calcium imaging signals of the cardiomyocytes in Hank’s balanced salt solution (HBSS) buffer that maintained a consistent extracellular calcium ion concentration of ∼1.2 mM. The procedure we used for recording and analyzing calcium transients has been described in detail (44). Briefly, the Fluo-4 Calcium Imaging Kit, purchased from Thermo Fisher Scientific (Cat. No. 10489) includes Fluo-4 acetoxymethyl ester (Fluo-4 AM) as a cell-permeable fluorescent calcium indicator to detect calcium handling in CMs. The mouse CMs were cultured in 12-well dishes with the revised culture medium for 3 days and then exposed to 5 μM of Fluo-4 AM, a single wavelength fluorescent calcium indicator, at 37°C for 20 min in HBSS with 10 mM HEPES containing 0.05% Pluronic-127 to facilitate solubilization of the indicator (Thermo Fisher Scientific). Subsequently, CMs were washed and incubated at room temperature for 40 min prior to the experiments to de-esterify the intracellular Fluo-4 AM. Upon cleavage by intracellular esterases, Fluo-4 AM releases Fluo-4, which binds to calcium ions with a *K*_d_ value of 345 nM. Calcium transient traces were imaged at 490–550 nm at 37°C/5% CO_2_/90% humidity, which were obtained from the Fluo-4 emission signal on the inverted microscope stage of a confocal system (Zeiss LSM980) with full environmental control (temperature, CO_2_, humidity) after argon laser excitation at 488 nm acquired at a sampling rate of 50.86 frames per second (fps) for 90 s by software (Zen 3.0). The chosen sampling rate of 50.86 fps, corresponding to an ∼20 ms interval time between images at 128 × 128 pixels, is deemed sufficient for achieving acceptable temporal resolution in our calcium trace analysis according to Nyquist theorem (a periodic signal must be sampled at more than twice the highest frequency component of the signal). The fluorescent intensity *I*(*t*) of individual cells in the regions of interest was normalized by using the baseline intensity *I*_o_ and the peak intensity *I*_m_ to obtain the relative signal *R*(*t*)* = *[*I*(*t*) − *I*_o_]/[*I*_m_ − *I*_o_], which was used in calculating the oscillation parameters as described in Refs. 45 and 46 and shown in the results (Fig. 4*D*), including beat period (peak-to-peak time, ms), time-to-peak (TTP, ms), 30% and 80% calcium reuptake duration times (CaD30 time and CaD80 time, ms), and decay time (ms), respectively. In contrast to adult cardiomyocytes cultured, which typically require external stimuli to induce contractions, mouse neonatal cardiomyocytes cultured undergo a rapid dedifferentiation-redifferentiation cycle, leading to spontaneous beating within 24-h postplating. Our study specifically examines spontaneous calcium oscillation in neonatal CMs cultured without external stimulation as previously described by Ehler et al. (47).

### Heart Hematoxylin-Eosin Stain

Hematoxylin-eosin (H&E) staining was used to identify cardiac tissues and to provide information about the pattern, shape, and structure of cells in cardiac tissue samples. Neonatal mice were euthanized at the postnatal age of 14 days, and the mouse heart photos were captured using an AmScope Stereo Microscope (SM-1TSZZ-144S-10M). The hearts harvested were fixed in neutral-buffered 10% formalin, embedded into paraffin blocks, and sectioned into slices at a thickness of 4–6 µm. H&E-stained sections were examined under a light microscope (Olympus Upright Microscope, BX-61) equipped with a ×10 eyepiece lens, a high-power objective lens (×40), and an oil immersion objective lens (×100). The extracellular matrix (ECM), appearing more eosinophilic and staining pink, was grossly quantified using ImageJ. The process involved converting images to grayscale, creating binary images, and using segmentation algorithms to isolate ECM regions. The area of these regions in pixels was calculated and converted to physical units, allowing the determination of the ECM area fraction by dividing it by the total tissue area. A quantitative comparison of ECM area fractions (%) between the sertraline and saline groups (16 slices total, *n* = 8 for sertraline and *n* = 8 for saline groups) was performed.

### Echocardiography

The measurements were made in accordance with the American Society for Echocardiography Guidelines by operators who were blinded as to the nature of the mice. After sedation with midazolam (0.1 mg subcutaneous injection) (Almaject, NJ), the anterior thorax, abdomen, and pelvis were shaved. This dosage of midazolam did not cause a significant decrease in heart rate and did not elicit any observable abnormal behaviors in the mice, except for the intended sedation. Aligning with the existing literature, it has been reported that deeper sedation achieved with a higher dose of midazolam (∼0.2 mg subcutaneous injection) similarly does not result in a significant reduction in heart rate (48). The mouse was grasped by the nape of the neck and cradled in the recumbent position in the imager’s hand. The warmed gel was applied to the ultrasound probe to the area of interest for optimizing the acoustic interface. The echocardiographer obtained all images and measurements without knowledge of the genotypes of the animals. Mouse echocardiograms were performed using a 40 MHz linear array probe coupled to a Vevo 2100 imaging system (VisualSonics, Toronto, ON, Canada) as described previously (18, 19, 49). After confirmation of optimal short-axis or long-axis orientation, a video clip spanning 1 s was digitized and stored offline for later quantitative analysis. At relevant heart rates the procedure captures 10–12 cardiac cycles per video clip for adult hearts.

Maternal-fetal echocardiograms were performed in pregnant mice. The transducer was placed directly on the maternal anterior pelvic wall and maneuvered to visualize each fetus, and the fetal heart was visualized via a short axis view. Blood flow velocities were measured using color Doppler. Video image clips were acquired and stored as described earlier. A 1-s video clip typically captures 3–4 fetal cardiac cycles. Video image clips were analyzed by an investigator blinded to treatment conditions. Digital images were transferred to a high-resolution workstation. Quantitation was performed using software provided by the vendor (VisualSonics). After viewing the entire image video clip, an investigator selected the single cardiac cycle with the clearest depiction of cardiac motion, and which conformed best to standard short-axis or long-axis orientation, respectively, for quantitative analysis. The procedures produce a final on-screen magnification of ×8–22, depending on the acquisition field of view.

For adult mice, the echocardiography images of the endocardial and epicardial area, in end-diastole and end-systole, respectively, were used in the parameter calculations. Maternal and fetal heart rates and beats per minute (HR, beats/min), maternal uterine and umbilical artery flow velocity (UmV and UtV, mm/s), maternal artery and umbilical artery resistance index (Um RI and Ut RI), fetal crown rump length (CRL, mm), placental thickness (mm), and left ventricle (LV) thickness were measured. The ejection fraction (EF, %), mass (M, mg), stroke volume (SV, µL), and cardiac output (CO, mL/min) were calculated using the biplane area-length method for computing ventricular volumes. Detailed descriptions and calculations of the echocardiography parameters are provided in the Supplemental material (https://doi.org/10.6084/m9.figshare.26360191.v1).

### Measurements of miRNA Expression

SSRIs bind to the 5-HT transporter (SLC6A4), inhibiting 5-HT reuptake, which, in turn, affects the signaling activity of 5-HT receptors (HTR2A and HTR2B). We quantified the expression levels of miRNAs targeting their corresponding mRNAs, which were identified based on our prior investigations in SSRI-exposed mice, along with online prediction tools including miRGate (50), miRWalk (51), and miRDB (52, 53). In addition, we included miRNAs documented in the published literature on cardiovascular disease and depression in our analysis (54–64). The detailed information of the selected miRNA candidates is shown in Supplemental Table S1 and Figs. S1 and S2 (https://doi.org/10.6084/m9.figshare.26360191.v1).

Frozen tissue was homogenized and RNA was extracted using TRIzol (Invitrogen) as described by the manufacturer, including chloroform phase separation on ice, isopropanol precipitation, and followed by 75% ethanol wash. Extracted RNAs were DNAse-treated (Ambion’DNase Treatment Kit, Thermo Fisher Scientific) to remove possible DNA contamination. miRNAs were enriched and purified using miRNeasy Kits (QIAGEN) according to the manufacturer’s protocol. The first-strand cDNA was synthesized using the miRCURY LNA RT Kit (QIAGEN) according to the protocol provided by the manufacturer. miRCURY LNA miRNA Custom PCR Panels (QIAGEN) were used to detect the miRNAs of 84 candidates with references and controls in 96-well plates using the miRCURY SYBR Green Master Mix (QIAGEN) in a CFX96 Real-Time System (Bio-Rad) according to the manufacturer’s protocol.

### qPCR for mRNA Expression in Mouse Hearts

qPCR was used to quantitate mRNA levels in the cardiac samples. In brief, total RNAs were extracted as aforementioned, and the quality and quantity of the RNA samples were checked using NanoDrop 2000 (Thermo Fisher Scientific) measured at 230, 260, and 280 nm. The RNAs were reverse transcribed into cDNA using the AffinityScript^M^ QPCR cDNA Synthesis Kit (Agilent Technologies) according to the instructions of the manufacturer. qPCR was carried out using the Brilliant SYBR Green QPCR Master Mix (Agilent Technologies) and primers (Supplemental Table S2) in the CFX96 Real-Time System (Bio-Rad). Gene expression values were normalized to the expression of a house-keeping gene (*Gapdh*). *Gapdh* is a commonly used reference gene to normalize the sample loadings in qPCR analysis, but the use of this reference has been challenged based on controversial findings for myocardium analysis (65, 66). By using geNorm analysis (https://genorm.cmgg.be/), we could verify stable expression of *Gapdh* mRNA in cardiac tissues with sertraline exposure versus saline [see Supplemental Fig. S3 (87, 88); see https://doi.org/10.6084/m9.figshare.26360191.v1]; based on this, we chose *Gapdh* as the normalization reference gene for this study. Relative expression was determined by the ΔΔCT method.

### Western Blotting for Protein Expression in Mouse Hearts

Western blotting was used to identify proteins in the cardiac sample. Detailed descriptions of the measurement and analysis of the Western blotting procedure are provided in the Supplemental material (https://doi.org/10.6084/m9.figshare.26360191.v1). Briefly, mouse hearts were harvested, washed with PBS, and homogenized in lysis buffer. Lysates were vortexed, sonicated on ice, and protein concentration was determined by the Bradford method (Bio-Rad Protein Assay kit). Proteins (50 µg) were denatured with 2× gel SDS loading buffer at 95°C for 5 min, separated on a 7.5% SDS-Tris-HCl polyacrylamide gel, and transferred to a nitrocellulose membrane. The membrane was blocked with 5% nonfat milk in TTBS, incubated with primary antibodies overnight (Supplemental Table S3), and then with horseradish peroxidase (HRP)-conjugated donkey anti-rabbit IgG for 1 h at room temperature. Protein detection was performed using an enhanced chemiluminescence system (Pierce) and X-ray film exposure. Membranes were stripped and re-probed with anti-GAPDH antibody (Sigma) for loading normalization. The specificity of the antibodies was validated using HEK293 cells as negative controls and mouse brain tissue as positive controls (Supplemental Figs. S4 and S5; see https://doi.org/10.6084/m9.figshare.26360191.v1).

For band density analysis, ImageJ was used. Images were converted to 8-bit mode, and the Rectangular Selections tool was used to designate each lane. Peak profiles were plotted for each band, highlighted with the Wand tool, and measurements were recorded for calculating relative density. This approach guarantees a meticulous analysis of Western blot band density with ImageJ, enabling accurate comparisons in quantitative protein expression studies (69).

### Statistical Analysis

Data are presented as means ± SE for continuous variables, and specific sample sizes are denoted in results. The effects of sertraline exposure versus saline controls were evaluated by unpaired two-tailed *t* test for two groups, and two-way ANOVA with Tukey’s multiple comparisons test for more than two groups when the data were normally distributed (Shapiro–Wilk test, *P* > 0.05). When the data were not normally distributed (Shapiro–Wilk test *P* < 0.05), the nonparametric Mann–Whitney test was used to assess statistical significance between the sertraline and saline groups for specific sex. Asterisks denote significance levels: *P < 0.05, **P < 0.01, and ***P < 0.001. Statistical analysis was performed using Prism 9.4.1 (GraphPad Software, Boston, MA).

## RESULTS

### Sertraline Exposure Decreased Pup Growth

The gestation periods exhibited no significant difference between the mouse groups subjected to either sertraline or saline treatments, ranging from a minimum of 18 days to a maximum of 21 days. Specifically, the durations were 19.5 ± 0.9 days for the sertraline exposure group and 19.1 ± 0.8 days for the saline group (*P* > 0.05).

In our model of chronic serotonin exposure, we treated pregnant female mice with sertraline or with saline (Fig. 1). In total between both treatment groups, 46 pregnant female C57BL/6J mice (age 10 wk) were used in this exposure model. Dam weights at the initiation of breeding were similar (20.17 ± 0.23 g) between the sertraline-treated and saline-vehicle control groups. The average litter size for all dams was seven pups (range 5–9). There were 137 pups (20 litters) in the saline group and 206 pups (26 litters) in the sertraline-treated group. There was no significant difference in the survival rate between the groups, which was 92%–93% (Supplemental Fig. S6; see https://doi.org/10.6084/m9.figshare.26360191.v1). There were no significant differences between the body weights of maternal mice with saline and with sertraline treatments (Supplemental Fig. S7; see https://doi.org/10.6084/m9.figshare.26360191.v1). There was no visible variance in food intake volume between those two groups of mice.

**Figure 2.**
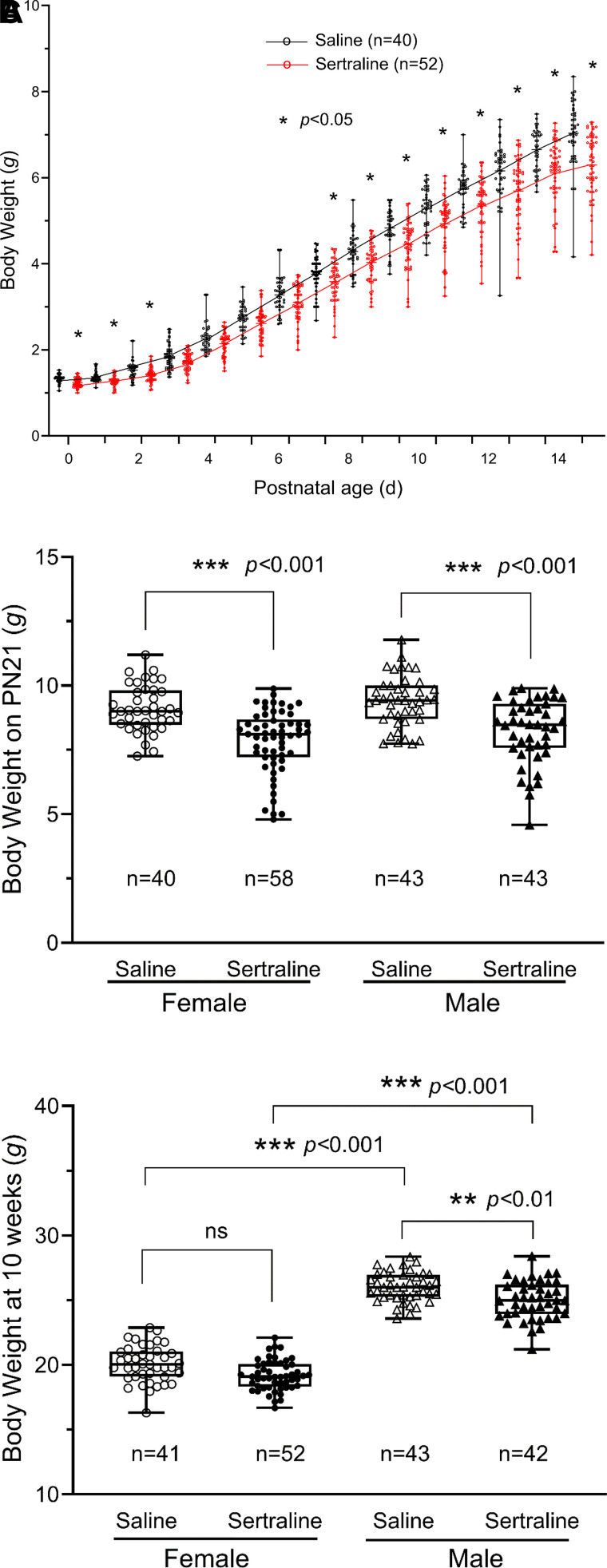
Sertraline exposure reduced body weights. *A*: individual scatter plots of body weights from two randomly selected mice per litter (plotting mean with range) were graphed in relation to postnatal age (*PN 0*–*14*) with saline (black circles) vs. sertraline (red circles). Box-plot of body weights (displaying min to max with all points) on *PN day 21* (*B*) and at 10 wk of age (*C*), presenting data of saline (Female: open circles, Male: open triangles) vs. sertraline (Female: filled circles, Male: filled triangles). The figures presented the number of pups in each group. The significance between the sertraline and saline groups was determined through two-way ANOVA with Tukey’s multiple comparisons test. *P < 0.05, **P < 0.01, ***P < 0.001.

Individual body weights of randomly chosen two mice per litter from all pups of saline group (137 pups/20 litters) and sertraline group (206 pups/26 litters) were used for the analysis and presented in Fig. 2*A*, which was similar to that of all data points (Supplemental Fig. S8; see https://doi.org/10.6084/m9.figshare.26360191.v1). Figure 2*A* shows that sertraline exposure significantly decreased pup body weight on *postnatal* (*PN*) *days 0*–*2*, and *8*–*14*. The sertraline-exposed group continued to show a significantly lower body weight than the saline group [female: sertraline 7.84 ± 0.16 (*n* = 58) vs. saline 9.03 ± 0.17 g (*n* = 40), *P* < 0.001; and male: sertraline 8.23 ± 0.19 (*n* = 43) vs. saline 9.39 ± 0.15 g (*n* = 43), *P* < 0.001] on *PN day 21*, as shown in Fig. 2*B*. At *PN day 21*, no sex-dependent difference was observed (*P* > 0.05). When the mice matured to 10 wk of age (Fig. 2*C*), as expected, the body weights became sex-dependent with males heavier than females (*P* < 0.001). The female body weights were not significantly lower in the sertraline-exposed group compared with the saline group (19.19 ± 0.16 g vs. 20.07 ± 0.22 g, *P* = 0.11). Male body weights were significantly lower in the sertraline-exposed group (24.99 ± 0.23 vs. 26.03 ± 0.17, *P* < 0.01).

**Figure 3.**
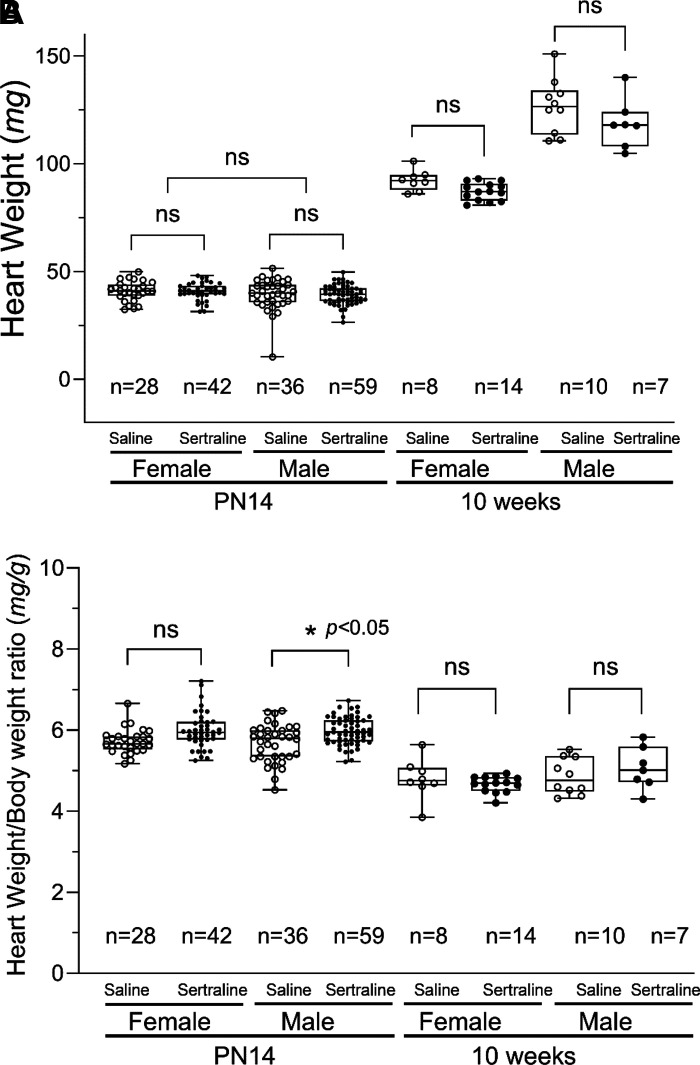
The effects of sertraline exposure on mouse heart weights at *PN14* and 10 wk. Box-whiskers plot of heart weight (*A*), and Box-whisker plot of heart/body weight ratios (*B*). Individual data were plotted with min to max and show all points for saline (○) vs. sertraline (●). The figures displayed the pup count for each group. Significance between the sertraline and saline groups was established using two-way ANOVA with Tukey’s multiple comparison test. *P < 0.05.

In Fig. 3*A*, heart weights on *PN day 14* were similar in sertraline compared with saline-exposed mice (female: 40.90 ± 0.09 mg vs. 40.92 ± 0.85 mg, *P* > 0.05; and male: 39.35 ± 0.58 mg vs. 39.44 ± 1.20 mg, *P* > 0.05). When sertraline-exposed pups were raised to adulthood, we found that these adult mice (10 wk old) showed a sex-dependent alteration. Sertraline exposure decreased female heart weights compared with saline, but this difference was not statistically significant (87.20 ± 1.09 mg vs. 92.31 ± 1.70 mg, *P* > 0.05). Male heart weights were decreased with sertraline exposure but not significantly (118.69 ± 4.33 mg vs. 126.67 ± 3.99 mg, *P* > 0.05). The heart weights were normalized by the body weights and the ratios obtained are shown in Fig. 3*B*. The ratios on P*N day 14* were higher in sertraline versus saline due to the reduced body weights resulting from sertraline exposure. For females, this increase was not significant (5.99 ± 0.07 mg/g vs. 5.72 ± 0.06 mg/g, *P* > 0.05), whereas for males, it was significant (5.98 ± 0.04 mg/g vs. 5.67 ± 0.08 mg/g, *P* < 0.05). The sex difference in relative heart weight was nullified in the adult mice by the decrease in both heart weight and body weight by sertraline exposure, and the heart/body weight ratios became similar between sertraline and saline groups at 10 wk of age (female: 4.67 ± 0.05 mg/g vs. 4.80 ± 0.18 mg/g, *P* > 0.05; and male: 5.06 ± 0.20 mg/g vs. 4.86 ± 0.14 mg/g, *P* > 0.05).

### Sertraline Exposure Slowed Ca^2+^ Oscillations in Neonatal Mouse Cardiomyocytes

**Figure 4.**
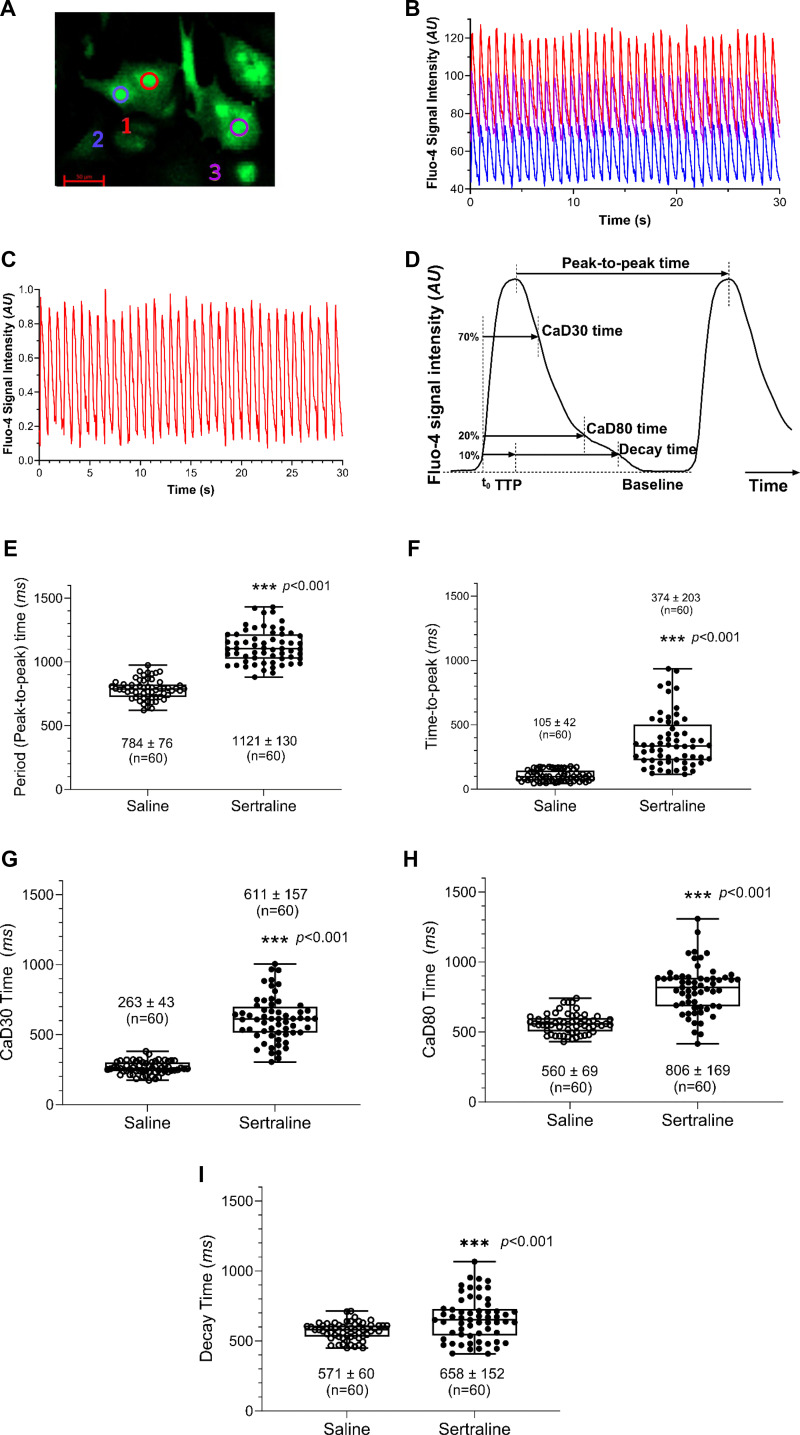
Sertraline affected Ca^2+^ oscillations in neonatal mouse cardiomyocytes (CMs). *A*: Fluo-4 Ca^2+^ indicator loaded in CMs (three cells are marked). *B*: raw Ca^2+^ oscillation signal traces from three cardiomyocytes obtained in a single sampling. The colors assigned (red, blue, and pink) correspond to the calcium signal traces of cell-1, cell-2, and cell-3 in *A*, respectively. *C*: the raw signal of cell-1 (red) was normalized to maximum. *D*: After setting reference initial time, to 10% of the Ca^2+^ upstroke to peak, the parameters characterizing the calcium transient dynamics of Ca^2+^ oscillation were extracted from the Fluo-4 signal (calcium transient) traces. They include period (peak-to-peak time), representing the duration of one complete oscillation (ms) (*E*); time-to-peak (TTP), the time from *t*_o_ to the peak of the oscillation (ms) (*F*); CaD30 time (the duration time from *t*_0_ to 30% of Ca^2+^ extrusion), characterizing a specific phase of the oscillation (ms) (*G*); CaD80 time (duration time from *t*_0_ to 80% of Ca^2+^ extrusion), capturing another phase of the oscillation (ms) (*H*); and decay time (the time from peak to 90% of Ca^2+^ extrusion), indicating the duration of the declining phase of the oscillation (ms) (*I*). *E*–*I* were graphed using box-plot (displaying min to max with all points), with ○ for saline, ● for sertraline, respectively. A total of 60 cells were counted, obtained by pooling hearts from three newborn mouse pups in each group from separate litters. Values in the figure were in means ± SD. Significance between the sertraline and saline groups was determined through an unpaired, two-tailed *t* test. ***P < 0.001.

The effects of in utero and neonatal sertraline exposure on calcium handling in neonatal cardiomyocytes were assessed quantitatively from the fluorescent intensity waveform of Fluo-4 calcium transients in cardiomyocytes (CMs) as shown in Fig. 4. The CM cells were cultured in 12-well dishes, and the view of the field in each dish was randomly chosen to locate the target cells. We examined multiple cells in the experiments, with the number of cells per sampling varying from 1 to 10, resulting in a total of 60 cells across 13 experiments for each group, available for subsequent analysis. The calcium signals from multiple cells exhibited synchronized oscillations, highlighting a consistent response pattern among the sampled cells. Sertraline exposure led to a significant increase in the beat periods from 784 ± 76 ms to 1,121 ± 130 ms (*P* < 0.001). Sertraline also significantly extended other characteristic oscillation times (*P* < 0.001) including time-to-peak (TTP) from 105 ± 42 ms to 374 ± 203 ms, CaD30 time (30% calcium reuptake duration time) from 263 ± 43 ms to 611 ± 157 ms, and CaD80 time (80% calcium reuptake duration time) from 560 ± 69 ms to 806 ± 169 ms, respectively. Importantly, calcium handling in CMs is contingent upon the beat period, representing time from peak to peak. The reuptake duration times, including CaD30 and CaD80, are directly impacted by the beat period of the cells. The heightened beat period noted in sertraline-exposed CMs would consequently extend these other parameters.

### Gene Expression in Hearts Relevant to Calcium Oscillation and Cardiac Function

**Figure 5.**
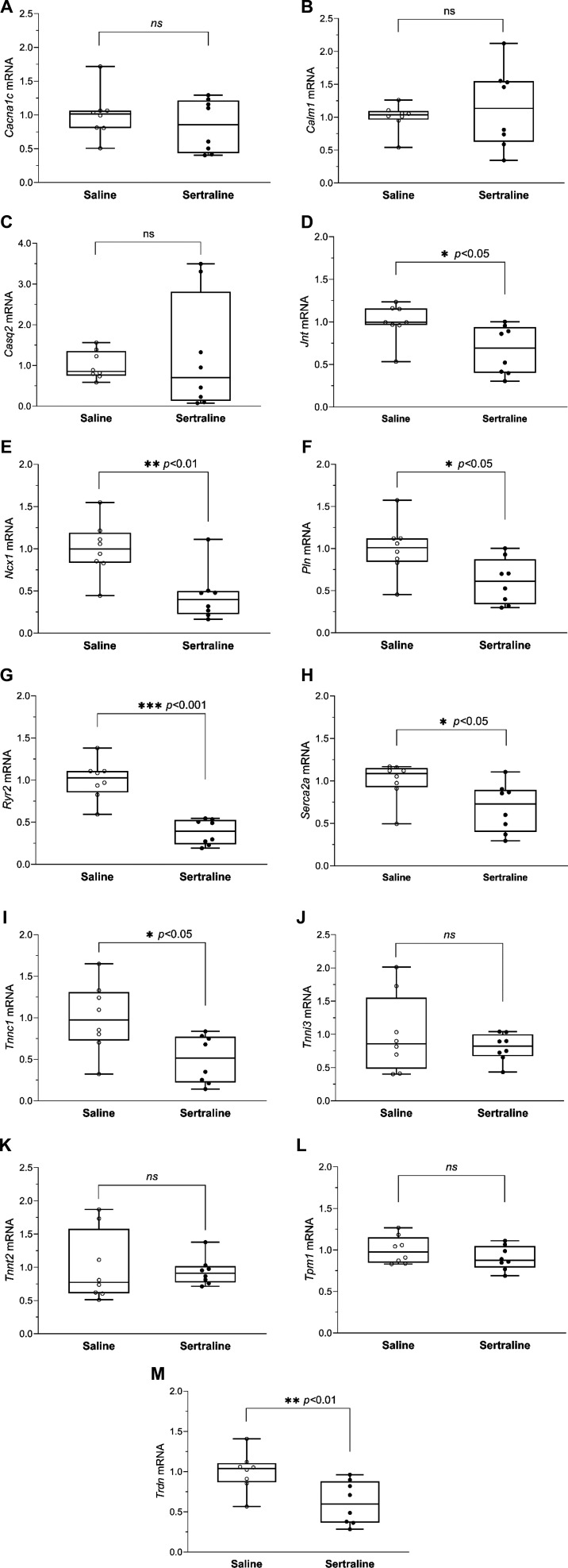
Effect of sertraline on cardiac expression of calcium signaling genes. Hearts were harvested on *postnatal* (*PN*) *day 14* from mouse pups treated with either sertraline or saline (*n* = 8 each group). Relative mRNA levels of key players regulating cardiac muscle contraction and relaxation within the intricate cardiomyocyte system, as determined by qPCR: *Cacna1c* (*A*), *Calm1* (*B*), *Casq2* (*C*), *Jnt* (*D*), *Ncx1* (*E*), *Pln* (*F*), *Ryr2* (*G*), *Serca2a* (*H*), *Tnnc1* (*I*)*, Tnni3* (*J*)*, Tnnt2* (*K*)*, Tpm1* (*L*), and *Trdn* (*M*). Data are graphed as box plots (displaying min to max with all points) with ○ for saline, and ● for sertraline, respectively. Significance testing between the sertraline and saline groups was conducted using an unpaired, two-tailed *t* test. *P < 0.05, **P < 0.01, ***P < 0.001.

Critical components within the intricate cardiomyocyte system regulate calcium signal oscillation, cardiac muscle contraction, and relaxation (70, 71). To examine the effects of sertraline on these processes, we analyzed mRNA levels of key genes involved in calcium handling and cardiac function in mouse hearts from sertraline or saline-treated pups (*n* = 8/group, *PN day 14*). The genes studied included *Cacna1c*, *Calm1*, *Casq2*, *Jnt*, *Ncx1*, *Pln*, *Ryr2*, *Serca2a*, *Tnnc1, Tnni3, Tnnt2, Tpm 1,* and *Trdn*. Detailed functions of these genes are provided in the Supplemental material (https://doi.org/10.6084/m9.figshare.26360191.v1). Our results showed that mRNA levels of *Cacna1c*, *Calm1*, *Casq2*, *Tnni3, Tnnt2,* and *Tpm1* (Fig. 5, *A–C* and *J*–*L*) remained unchanged with sertraline exposure compared with saline. However, sertraline treatment significantly decreased the mRNA levels of seven genes including *Jnt*, *Ncx1*, *Pln*, *Ryr2*, *Serca2a*, *Tnnc1,* and *Trdn* (Fig. 5, *D–M*).

### Sertraline Exposure Affected Mouse Heart Histology

**Figure 6.**
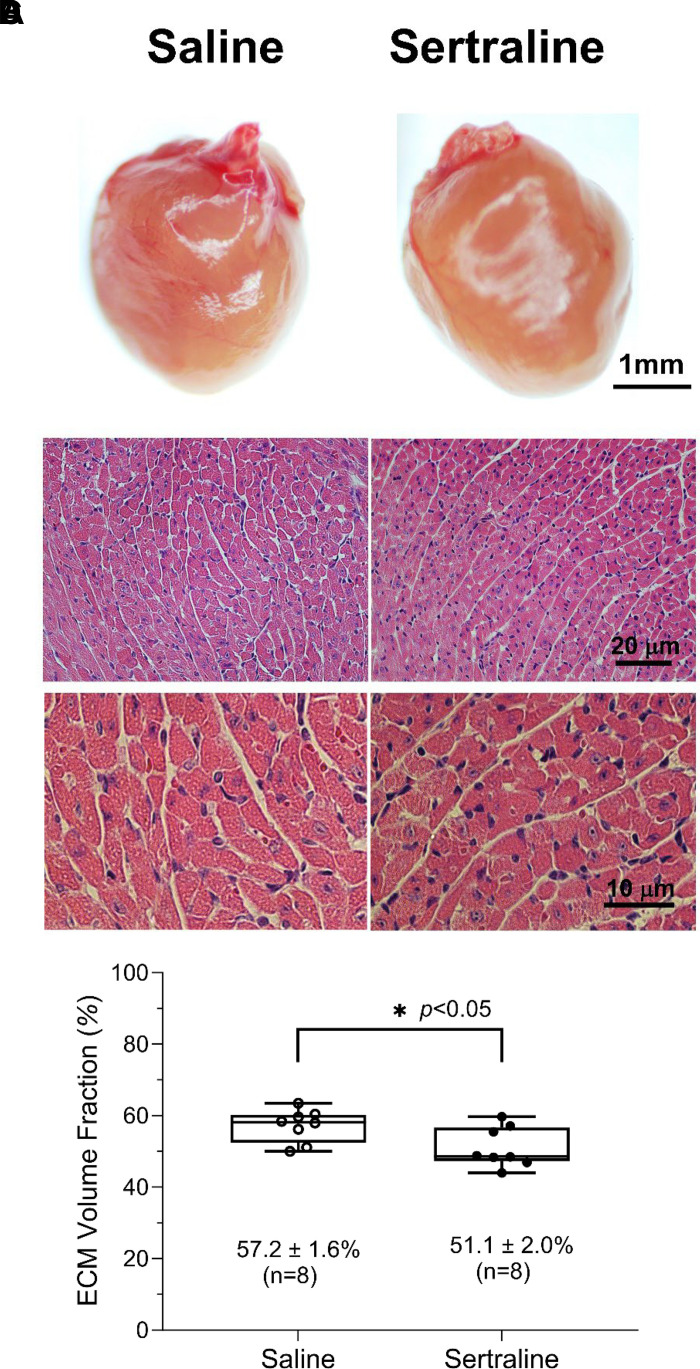
Sertraline exposure affected the cardiac extracellular matrix. Hearts were harvested on *postnatal* (*PN*) *day 14* from mouse pups treated with either sertraline or saline. *A*: mouse heart photos were captured using an AmScope Stereo Microscope (SM-1TSZZ-144S-10M) and software. Scale bar = 1 mm. Hematoxylin and eosin (H&E) stained sections were examined under a light microscope (Olympus BX-61) equipped with a ×10 eyepiece lens, along with a high-power objective lens (×40), scale bar = 20 µm (*B*); and with an oil immersion objective lens (×100) (*C*), scale bar = 10 µm. Nucleus is blue and cytoplasm is red, and collagen fibers show a varying red color. *D*: quantitative comparison of extracellular matrix (ECM) area fractions (%) between the sertraline and saline groups (16 slices total, *n* = 8 for sertraline and *n* = 8 for saline groups). Values of ECM area fractions were in means ± SE (%) in the graph. Significance between the sertraline and saline groups was determined through an unpaired, two-tailed *t* test. *P < 0.05.

Figure 6 displays the morphology and histology of neonatal hearts subjected to in utero and neonatal sertraline exposure, contrasting them with neonatal hearts exposed to saline. Both sets exhibited grossly normal major structures and histology. No defects, inflammatory cell infiltration, fibrosis, or necrosis of myocardial cells were identified in either group. In addition, no significant structural or histologic disparities were observed between the hearts of sertraline-exposed (in utero + neonatal) and saline control adult mice (10 wk old, data not shown), aligning with our prior observations (18). However, an examination of extracellular matrix (ECM) area fractions (%) revealed a reduction in ECM under sertraline treatment, as evident in the H&E staining images of mouse cardiac tissues (Fig. 6*C*). Quantitative assessments based on 16 slices provided statistical insights into the impact of sertraline on the cardiac tissue microenvironment (Fig. 6*D*). However, ECM may be over-represented as we did not specifically delineate collagen content which also may exhibit shades of pink or red.

### Sertraline Exposure Altered miRNA Profiles to Regulate Gene Expression in Hearts

**Figure 7.**
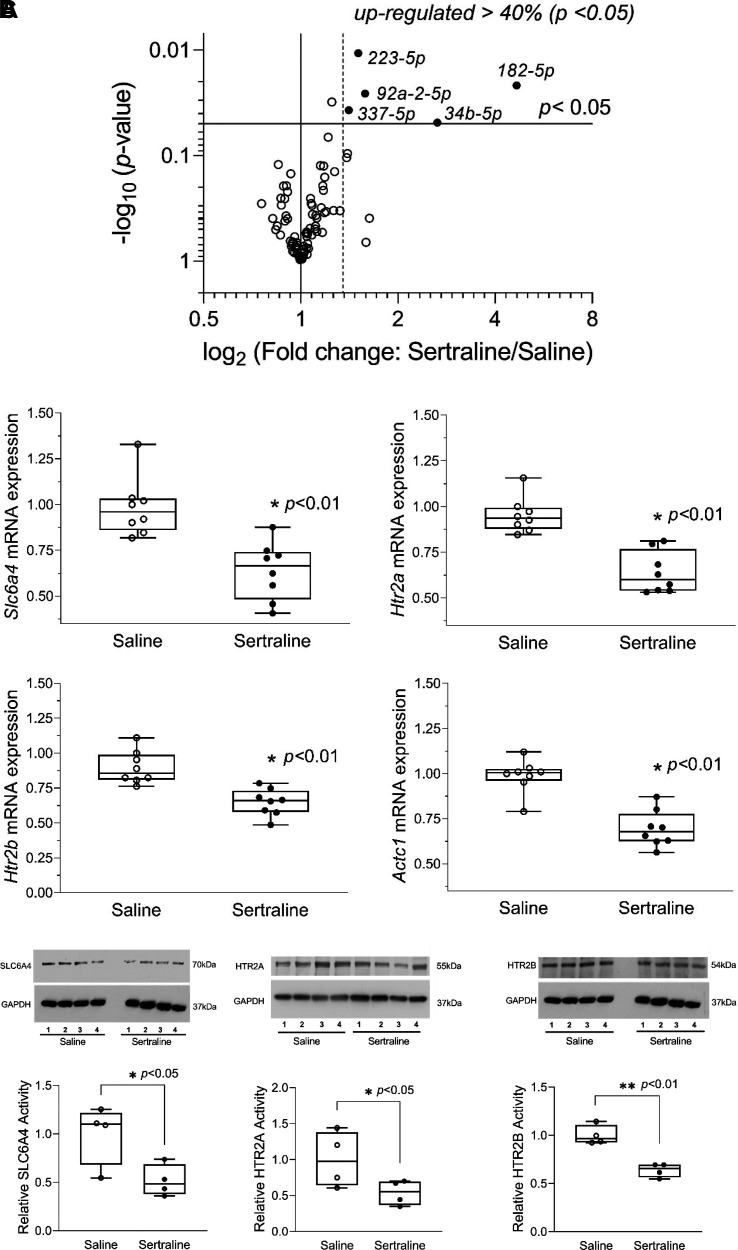
Sertraline exposure altered microRNAs (miRNAs) that epigenetically regulate gene expression in hearts. Hearts were harvested on *postnatal* (*PN*) *day 14* from mouse pups treated with either sertraline or saline. *A*: volcano plot of miRNAs using log-plotting of *P* values against fold change, to visualize the results of a differential gene expression analysis. *B*: the relative mRNA expression levels of *Slc6a4, Htr2a,* and *Htr2b* in mouse hearts with sertraline exposure were significantly decreased relative to the saline control group (*n* = 8/group, *P < 0.01). *C*: the corresponding protein expression changes in mouse hearts were consistent with the effects on mRNA expression (*n* = 4/group, *P < 0.05, **P < 0.01). B and *C* were graphed using box-plot (displaying min to max with all points) with ○ for saline, and ● for sertraline, respectively. Of note, Slc6a4 and HTR2B were obtained using the same membrane. Statistical significance between the sertraline and saline groups was determined through an unpaired, two-tailed *t* test. *P < 0.05, **P < 0.01.

miRNAs are small, noncoding RNA molecules that play a crucial role in regulating gene expression by binding in a sequence-dependent fashion to one or more specific messenger RNAs (mRNAs) and inhibiting the target’s translation into protein. We evaluated whether sertraline exposure altered *1*) the levels of 84 miRNAs that are predicted to target the myocardial expression of genes involved in serotonin signaling (*Slc6a4, Htr2a, and Htr2b*) on *PN day 14* and *2*) the mRNA levels of select cardiomyocyte markers (*Actc1, Myl7, Nppa, and Npr3*). These 84 miRNAs are shown in Supplemental Table S1 (https://doi.org/10.6084/m9.figshare.26360191.v1). Five miRNAs were upregulated by 40% after sertraline exposure relative to the saline-treated group (*n* = 4/group, *P* < 0.05) as shown in Fig. 7*A* (*Htr2a: miR-34b-5p, miR-182-5p; Htr2b: miR-223-5p, miR-92a-2-5p, miR-337-5p; Slc6a4: miR-223-5p, miR-92a-2-5p, miR-182-5p*). Among them, *miR-182-5p* was elevated 4.7-fold.

The levels of the corresponding mRNA targets, *Slc6a4, Htr2a*, and *Htr2b,* were significantly decreased in mouse hearts with sertraline exposure versus saline (normalized to 1.00), as shown in Fig. 7*B* (*Slc6a4:* 0.67 ± 0.04, *Htr2a*: 0.72 ± 0.03, *Htr2b* 0.73 ± 0.05, *n* = 8/group, *P* < 0.01), consistent with inhibitory effects of the upregulated miRNAs. The results of the qPCR analysis of mRNA expression were further confirmed by analysis of protein expression by Western blotting. SLC6A4 protein expression was decreased by sertraline exposure from 1.00 ± 0.16 to 0.51 ± 0.08 (*n* = 4/group, *P* < 0.05); HTR2A from 1.00 ± 0.17 to 0.54 ± 0.09 (*n* = 4/group, *P* > 0.05); HTR2B from 1.00 ± 0.05 to 0.64 ± 0.04 (*n* = 4/group, *P* < 0.01) (Fig. 7*C*). α Cardiac actin, ACTC1, is the major protein of the thin filament in cardiac sarcomeres and is responsible for muscle contraction and generation of force to support the pump function of the heart. *Actc1* mRNA was decreased in sertraline-exposed mice (Fig. 7*B*, *bottom right quadrant*).

### Cardiovascular Function in Mice

Maternal sertraline exposure did not alter umbilical or uterine artery blood flow. Slightly higher values of umbilical and uterine artery flow velocities were observed in the pregnant mice with sertraline exposure but were not significantly different from the values in the saline control dams (*P* > 0.05), as shown in Supplemental Table S4 (https://doi.org/10.6084/m9.figshare.26360191.v1). The sertraline-exposed fetuses showed lower heart rates but were not statistically significant (153 ± 19 beats/min vs. 175 ± 16 beats/min).

**Figure 8.**
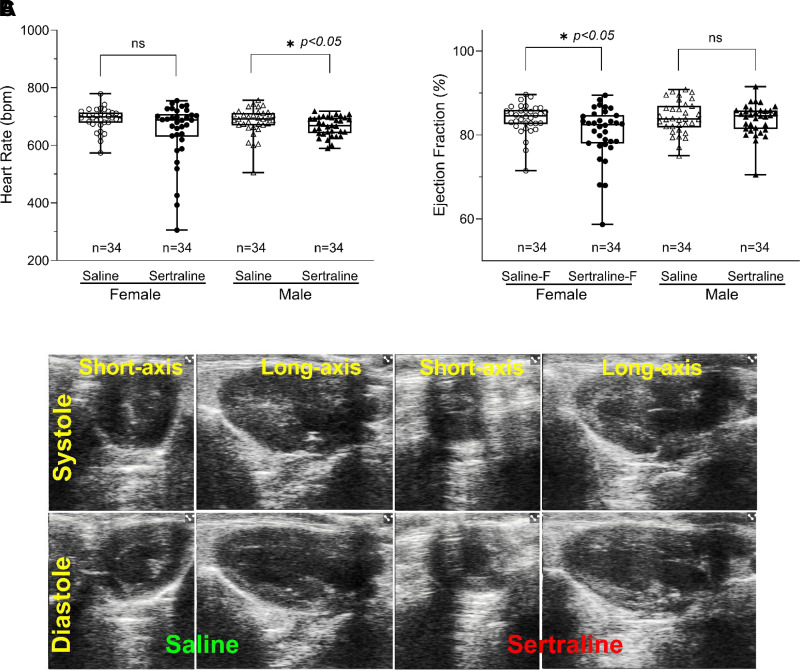
Cardiac function analysis using echocardiography for adult mice with perinatal plus neonatal sertraline exposure vs. saline. *A*: heart rate. *B*: ejection fraction. *C*: representative echocardiographic images. A and *B* were graphed using box-plot (displaying min to max with all points) with ○ for female saline and Δ for male saline, and ● for female sertraline and ▴ for male triangles, *n* = 34 for each group, respectively. Statistical significance between the sertraline and saline groups was determined through an unpaired, two-tailed *t* test. *P < 0.05.

Echocardiograms on adult mice at 10 wk of age (that had been exposed to sertraline perinatally as per Fig. 1) showed that sertraline exposure led to a decreased heart rate in male mice (saline 683 ± 8 beats/min vs. sertraline 666 ± 6 beats/min, *n* = 34/group, *P* < 0.05) and ejection fraction in female mice (saline 83.9 ± 0.6% vs. sertraline 80.6 ± 1.1%, *n* = 34/group, *P* < 0.05) (Fig. 8, *A* and *B*). Representative images of short-axis and long-axis views in systole and diastole are shown in Fig. 8*C*. No other cardiac functional data—including left ventricle thickness, mass, volume/mass, stroke volume, cardiac output, and fractional shortening—showed significant alterations by sertraline versus saline (Table 1).

**Table 1.** Echocardiogram data from mice at 10 wk of age

	Saline		Sertraline	
Parameter	Female	Male	Female	Male
*n*	34	34	34	34
LV thickness, mm	0.64 ± 0.01	0.64 ± 0.01	0.63 ± 0.01	0.68 ± 0.02
Heart rate, beats/min	691 ± 7	683 ± 8	649 ± 18*	666 ± 6
LV mass, mg	57.40 ± 1.39	66.71 ± 1.53	55.21 ± 2.00	67.13 ± 1.95
LV EDV/mass, µL/mg	0.36 ± 0.01	0.40 ± 0.01	0.39 ± 0.02	0.39 ± 0.02
Stroke volume, µL	17.22 ± 0.63	22.91 ± 1.13	17.55 ± 1.18	22.30 ± 1.38
Cardiac output, mL/min	11.88 ± 0.43	15.56 ± 0.74	10.91 ± 0.57	14.84 ± 0.91
Ejection fraction, %	83.88 ± 0.62	84.19 ± 0.67	80.61 ± 1.10*	83.75 ± 0.64

Data are means ± SE. Unpaired and two-tailed *t* tests were used to compare the parameters between sertraline group and saline group for each sex. EDV, end-diastolic blood volume; LV, left ventricle.

* *P* < 0.05.

Outliers were observed in heart rate measurements (<500 beats/min) within the female group. To determine whether these outliers contribute to the reported statistical significance in heart rate (HR) and ejection fraction (EF) data, we conducted outlier tests on the HR and EF datasets of mouse groups using *Z*-scores [*Z* = (*X* − Mean)/(Standard Deviation)] with a threshold set at |*Z*| ≤ 2 (72). The results are presented in Supplemental Fig. S9 (https://doi.org/10.6084/m9.figshare.26360191.v1). The datasets showed a few outliers, and notably, an outlier in heart rate for a given mouse did not correspond to an outlier in EF for the same mouse. The analysis indicates that the statistical significance in both HR and EF remains unaffected by the presence of outliers in either variable.

Since we included all data points in Fig. 8, *A* and *B* and they are not normally distributed (Shapiro–Wilk test, *P* < 0.05), we used the nonparametric tests for the HR and EF data to focus on specific treatment effects within each sex and on comparisons between all four groups simultaneously. The latter analysis showed that the effects of sertraline versus saline on HR and EF in both sexes were no longer significant (*P* > 0.05), suggesting that the treatment effects are sex-specific and may be masked when analyzing all groups simultaneously (Supplemental Table S5, *A* and *B*; see https://doi.org/10.6084/m9.figshare.26360191.v1).

## DISCUSSION

Neuropsychiatric disorders such as depression contribute significantly to disease burden worldwide. Despite the significant effects of these disorders on public health, progress in understanding the pathophysiology of the disease and the discovery of novel therapeutics has been slow (39). For depression, clinical characteristics and disease severity vary widely among individuals with the disease. Depression is diagnosed based on a cluster of highly variable symptoms. In addition to depressed or irritable mood, depression includes cognitive symptoms (guilt, ruminations, suicidality), emotional symptoms (anhedonia), homeostatic symptoms (abnormalities in sleep, appetite, weight, energy), and psychomotor symptoms (agitation or retardation). Only a subset can be modeled and measured objectively in rodents. Even with the increasing ease of developing rodent and invertebrate models by genetic manipulation or other means, difficulties in modeling human disorders such as depression have not been averted.

Up to 20% of women suffer from depression during the perinatal period (73) and SSRIs are the most prescribed therapy. As highlighted in introduction, SSRI use is now estimated to be as high as 15% in pregnant women in the United States (1, 2). SSRIs are prescribed in pregnancy with the expectation that they promote maternal mental health, and thus provide a developmental health benefit to the fetus and the child. SSRIs have been associated with adverse fetal effects though interpretation of these findings has been clouded by confounding maternal depression (3–8). Due to the prevalence of SSRI usage in pregnancy, there is a growing body of research examining perinatal SSRI effects in animals during development and beyond birth (37, 38). Animal models thus can provide important insight into the biological actions of SSRIs and how SSRIs may contribute to abnormal development even in the absence of maternal depression. Given the associations between SSRI exposure with congenital heart disease, our interest is in how SSRIs influence heart development and future heart health.

In this study, we investigated the impact of sertraline exposure on the expression of miRNAs, modulation of gene expression, and impact upon postnatal cardiac phenotype. Our previous mouse studies, and our zebrafish study, suggested that sertraline exposure results in reduced cardiomyocyte numbers and that these SSRI effects were mediated via the serotonin signaling pathway including the 5-HT transporter (SLC6A4) and receptors (HTR2A and HTR2B) (19, 20). In this current study, we hypothesized that epigenetic modulation of the serotonin pathway genes by miRNAs would correlate with abnormal cardiac development and function.

miRNAs are small, noncoding RNA molecules that play a crucial role in regulating gene expression by binding in a sequence-dependent fashion to one or more specific messenger RNAs (mRNAs) and inhibiting the target’s translation into protein. miRNAs may also trigger mRNA degradation along with translational repression. miRNAs, as noncoding RNAs, constitute a key component in the distinct layers of epigenetic regulation that modulate cardiac prenatal development and postnatal maturation (9, 54–56, 74). miRNAs also play important roles in regulating the complex biological processes of different cardiovascular diseases. Though the involvement of cardiac miRNAs in the heart and in cardiac disease states has been studied, the associations between cardiac miRNAs and heart development and function are not as well described (74). 5-HT is one of the endogenous molecules known to affect the cellular miRNA profile.

A major finding of this study is that five miRNAs were upregulated in heart tissue in our sertraline exposure mouse model compared with saline-exposed controls. Those upregulated miRNAs include *miR-34b-5p* and *miR-182-5p* targeting *Htr2a*, *miR-223-5p, miR-92a-2–9, and miR-337-5p* targeting *Htr2b*, and *miR-223-5p, miR-182-5,* and *miR-92a-2-5p* targeting *Slc6a4*. Alterations of specific miRNAs have been reported to be associated with the regulation of cardiogenesis, congenital disorders in newborns, and adult cardiovascular disease (54–62, 75). Of note, four of the five upregulated miRNAs in our study play critical roles in heart disease. Overexpression of *miR-182-5p* has been associated with perturbations of heart morphology, and calcium handling, and with the onset of arrhythmias (58, 59). Furthermore, the downregulation of *mi-182-5p* reduced cardiac defects in a zebrafish model of Holt-Oram syndrome, an autosomal dominant disorder characterized by severe upper limb and cardiac malformations (58). Both *miR-182-5p* and *miR-223-5p* have been suggested as biomarkers for heart failure (59, 60). *miR-337-5p* plays a role in cardiac development and hypertrophy (61, 62). *miR-337-5p* was upregulated in the right ventricular outflow tract myocardium tissue obtained from infants with Tetralogy of Fallot (62). Finally, the overexpression of *miR-92a-2-5p* attenuates oxidative stress injury in cardiomyocytes (75). Our miRNA data as presented only demonstrate correlation and there may be other miRNAs correlated with the serotonin signaling pathway. The upregulated miRNAs were associated with decreased mRNA expression of the relevant genes (*Slc6a4*, *Htr2a*, and *Htr2b*), which was confirmed at the level of the expression of the corresponding proteins for SLC6A4 and HTR2B. The corresponding protein for HTR2A was not significantly reduced compared with saline (*P* > 0.05). Future experiments are needed to establish causality between our miRNA findings and the regulation of the serotonin signaling pathway.

In general, SLC6A4 transports 5-HT intracellularly, thereby limiting extracellular 5-HT and thus 5-HT receptor activity. A mouse model with *Slc6a4* deleted demonstrated increased myocardial fibrosis compared with nondeleted controls, thus suggesting that diminished SLC6A4 expression plays a role in the pathophysiology of cardiac disease (76). The 5-HT-induced myocardial fibrosis was increased in *Htr2b* null mice but not in *Htr2a* null or *Htr2a/2b* double null mice (76). 5-HT2B receptors are required for normal cardiac development (9, 21, 22). In surviving adult *Htr2b* null mice, echocardiography confirmed the presence of left ventricular dilation and decreased systolic function (22). Taken together, these observations suggest that *Htr2b* expression is more sensitive to sertraline exposure, whereas the effect on *Htr2a* could be variable as observed in our previous and present studies (19, 20). In our mouse model of sertraline exposure, the expression of the serotonin transporter gene (*Slc6a4*) and of the serotonin receptor genes *Htr2a* and *Htr2b* were reduced; this might be associated with the decreased heart rate and ejection fraction that is seen in adult female mice compared with the saline control mice. There were no other cardiac functional parameters that were altered, suggesting the exposure was limited and sex-dependent, but it is unclear whether the significant alterations in 5-HT signaling would be a potential risk upon further stress and if, under such stress, males would be affected as well as females.

One novel finding of this study is that sertraline exposure alters the calcium handling of neonatal mouse CMs, an important aspect in cardiac development. Calcium handling is a complex process that plays a critical role in cardiac arrhythmias due to the ion’s roles in electrical and contractile function. Abnormalities in calcium handling can result in changes in the strength and timing of heart muscle contractions and can contribute to the development of various heart conditions. Arumugasaamy et al. (77) reported a similar effect of SSRIs exposure on induced pluripotent stem cell derived-cardiomyocytes in a model of placenta-fetus. They observed elongated calcium handling, that is, modest increases in the period of calcium oscillations, in response to SSRIs. We found much stronger period elongations in the primary CMs in contrast to the iPSC CM line. These observations are all consistent with the results of Park et al. (34), who found that SSRIs inhibited L-type Ca^2+^ currents to prolong the duration of cardiac action potentials, in turn, leading to cardiac arrhythmias (78), due to the contributions of L-type Ca^2+^ currents to the electrical and contractile functions of the heart (79). Interestingly, overexpression of *miR-182-5p* (targets *Htr2a* mRNAs), as observed in our study, also results in altered calcium handling and arrhythmias (58).

We further investigated how sertraline exposure during development impacted the gene expression of several key players in calcium handling and cardiac function. We found that perinatal sertraline exposure decreased gene expression in cardiomyocytes of several genes including *Jnt, Ncx1, Pln, Ryr2, Serca2a, Tnnc1,* and *Trdn.* We did however not confirm these findings by measuring protein levels. The mRNA levels of *Tnnc1*, which encodes the troponin subunit responsible for binding calcium, were significantly decreased following sertraline exposure. The troponin-tropomyosin complex is critical for converting calcium signals into mechanical force in cardiomyocytes, thereby controlling the heart’s pumping action. However, the mRNA levels of other components of the complex, such as *Tnnt2*, *Tnni3* (the noncalcium binding troponins), and *Tpm1* (tropomyosin), were unaffected. This change in *Tnnc1* mRNA is particularly relevant because the corresponding protein directly interacts with calcium, whereas the other components have regulatory roles without directly binding calcium. Tagashira et al. (80) evaluated the acute effects of fluvoxamine treatment on caffeine- or ryanodine-induced Ca^2+^ release to the cytosol in rat hearts and found that fluvoxamine treatment significantly reduced ryanodine-induced Ca^2+^ release to the cytosol. Gilbert et al. (81) highlight that while cardiomyocytes rely on Ca^2+^ signaling for the core task as a contractile unit in the heart and Ca^2+^ dysregulation is a main contributor to the failure of the heart pump, Ca^2+^ signaling in cardiomyocytes is equally pivotal as a mechanism for regulating cardiomyocyte growth and physiologic remodeling. Further work is needed to understand the significance of our findings and specifically, if Ca^2+^ dysregulation has a larger picture in SSRI-associated heart defects.

Importantly, this study highlights some important differences from our prior work. First, we had seen variable effects on body weight following sertraline exposure in prior studies (18, 19, 42). In this current study, sertraline exposure significantly decreased pup’s body weights in the neonatal period, at the time of weaning, and this difference persisted into young adult life. These data are consistent with clinical data supporting lower body weight in infants born to mothers taking SSRIs (82). Part of this inconsistency may be related to a smaller sample size and the inability to detect subtle changes in weight (19). To address potential litter effects, we randomly chose two mice from each litter for studies during the neonatal period. Our findings were consistent with the limited sample and when we included every mouse in the litter, thus suggesting minimal to no litter effect. Our previous study did demonstrate that sertraline exposure led to significant decreases in the expression of cardiac *Htr2b* mRNA and HTR2B protein sampled from the mice at *PN day 21*, an effect that was confirmed at *PN day 14* in this study. However, the results in this study regarding effects on the expression of *Slc6a4* and *Htr2a* mRNAs did not match previous data (19). We interpret the discrepancies to be caused possibly by *1*) spatial differences in gene expression for left ventricles, which were the tissue source for qPCR in the previous study, versus whole hearts, the tissue source for mRNA quantitation in the present study, *2*) potentially stronger effects on *Htr2b* with sertraline, and *3*) stage differences (*PN day 14* vs. *PN day 21*) between the prior and current mRNA expression analyses. Because we examined mRNA and protein expression in the present study, finding both to show the same trend, we feel confident in the observed downregulation of the expression of *Slc6a4, Htr2b*, and *Htr2a.* Finally, we observed some differences in our cardiac phenotype. Of note, we observed higher heart rates in this study compared with prior studies (18, 19). One explanation is that mice were previously recorded in their home environment via radiotelemetry rather than while being restrained for echocardiography. In addition, we previously used isoflurane for sedation for all mouse echocardiograms. In this study, we used midazolam, which enhances the dominance of sympathetic activity in the cardiac autonomic nervous system during conscious sedation (83). It is possible both methodological differences contributed to the markedly higher heart rates seen in this study compared with our previous studies. As such, it will be difficult to make a direct comparison between the echocardiography results reported here and those in our prior studies. However, we did observe a reduction in ejection fraction in this study, similar to the reduced shortening fraction in our earlier study, though it was only observed in female mice in the current study and may not have meaningful clinical significance at baseline (19). Further studies should investigate whether these subtle differences are exacerbated with stress. Although specific phenotypic expression remains slightly variable across SSRI exposure models, the overall directionality of the effect and an enhanced risk of adult cardiac dysfunction following perinatal SSRI exposure have been consistently demonstrated.

It is important to highlight that our animal model is in the absence of maternal depression and is specific to only sertraline, not other SSRIs. Similar animal models are described in recent reviews highlighting the impact of perinatal SSRI exposure on body weight and neurodevelopment also in the absence of maternal depression (37, 38). We assume similar pregnancy hormones and changes associated with lactation would be present in both wild-type (WT) and sertraline-exposed mice though these were not evaluated. Sertraline is one of the safest antidepressants to use during lactation and has been extensively studied (84–87). A review by Pinheiro et al. (88) highlights that there is essentially no appreciable transfer of sertraline through breast milk. As such, we do not think the transfer of sertraline via breast milk had a meaningful impact on our results.

Finally, important limitations remain in our approach which merits mention. In the current experimental approach, causation between miRNA expression and physiologic endpoints has not been directly established. We focused only on spontaneous calcium handling results and the inclusion of responses to external stimuli would allow us to address rate-dependency of calcium imaging studies, thus establishing a better understanding of the dynamic changes in calcium regulation under physiologic and pathologic conditions. Furthermore, we have included outliers in female-treated mice, with heart rates approaching 300–350 beats/min, which may have influenced other physiologic endpoints.

In conclusion, our observations of epigenetically modulated alterations of the serotonin signaling pathway, potentially mediated by miRNAs, and of altered calcium handling and gene expression of key regulators in excitation-contraction coupling with sertraline exposure are novel findings. Future work needs to determine the causality between the upregulated miRNAs, serotonergic signaling, and physiologic endpoints to provide mechanistic pathways for these associations with the goal of discovering new knowledge on the specific biologic actions of SSRIs that may contribute to abnormal heart development. This work will ultimately help offer a better understanding for clinicians of how SSRIs may influence fetal development and potential modifications of current therapy.

## DATA AVAILABILITY

Data will be made available upon reasonable request.

## SUPPLEMENTAL MATERIAL

10.6084/m9.figshare.26360191.v1 Supplemental Tables S1–S5, and Supplemental Figs. S1–S9: https://doi.org/10.6084/m9.figshare.26360191.v1.

## GRANTS

This work is financially supported in part by National Institutes of Health Grants K08HL141528 and R01HL146363 (to S. E. Haskell) and S100D019941 to (R. M. Weiss).

## DISCLOSURES

No conflicts of interest, financial or otherwise, are declared by the authors.

## AUTHOR CONTRIBUTIONS

S.E.H. conceived and designed research; Y.L., E.K., and K.Z. performed experiments; Y.L. and S.E.H. analyzed data; Y.L., R.M.W., R.D.R., and S.E.H. interpreted results of experiments; Y.L. prepared figures; Y.L. drafted manuscript; Y.L., R.M.W., R.D.R., and S.E.H. edited and revised manuscript; Y.L., E.K., K.Z., R.M.W., R.D.R., and S.E.H. approved final version of manuscript.
